# Manifestations of antisocial behavior in older adults: A review of current literature

**DOI:** 10.1192/j.eurpsy.2024.704

**Published:** 2024-08-27

**Authors:** M. Arts, A. Videler, A. van Dam

**Affiliations:** ^1^Tranzo Scientific Center for Care and Welfare, Tilburg School of Social and Behavioral Sciences, Tilburg University, Tilburg; ^2^Research and Innovation, GGZ WNB Mental Health Institute, Halsteren; ^3^Mental Health Institute, Innovation and Quality, GGZ Breburg, Tilburg, Netherlands

## Abstract

**Introduction:**

Antisocial behavior in older adults is a problem for their relatives and health care providers Antisocial behavior may present differently in the older population which makes it more difficult to diagnose adequately and apply therapeutic interventions. This literature review provides an overview of diverse conceptualizations of antisocial behavior in older adults and the way it affects recognizability of diagnostic categories and the applicability of interventions.

**Objectives:**

To gain insight into the various manifestations of antisocial behavior in older adults.

**Methods:**

A systematic review design was performed. In this review, an extensive manual and electronic literature search was conducted for papers published from 1980 to 2023. For this purpose we used the electronic databases PubMed and Embase. The review will include empirical and quantitative studies of older adults with antisocial behavior.

**Results:**

The results from the literature indicate that antisocial behavior does probably not decrease with age nor the burden on their social environment. Rather, the manifestations of antisocial behavior change as this population ages. Personality disorders are determined by several dimensional trait domains. The domains which are highly predictive for antisocial behavior include antagonism and disinhibition.

**Conclusions:**

These findings challenge the notion of antisocial behavior decreasing with age. This review underscores the need to shift from traditional personality disorder categories to a dimensional trait perspective. Therefore, specific interventions are needed for older adults.

**Disclosure of Interest:**

None Declared

